# Translocation and potential neurological effects of fine and ultrafine particles a critical update

**DOI:** 10.1186/1743-8977-3-13

**Published:** 2006-09-08

**Authors:** Annette Peters, Bellina Veronesi, Lilian Calderón-Garcidueñas, Peter Gehr, Lung Chi Chen, Marianne Geiser, William Reed, Barbara Rothen-Rutishauser, Samuel Schürch, Holger Schulz

**Affiliations:** 1 Institute of Epidemiology, GSF-National Research Center for Environment and Health, Neuherberg, Germany; 2 Focus Network of Aerosols and Health, GSF-National Research Center for Environment and Health, Neuherberg, Germany; 3 National Health and Environmental Effects Research Laboratory, Neurotoxicology Division, US Environmental Protection Agency, RTP, NC, USA; 4 Instituto Nacional de Pediatría, Mexico City 14410, Mexico; 5 The Center for Structural and Functional Neurosciences, University of Montana, Missoula, MT 59812, USA; 6 Institute of Anatomy, University of Bern, 3012 Bern, Switzerland; 7 New York University School of Medicine, Department of Environmental Medicine, Tuxedo, NY, USA; 8 Department of Pediatrics and Center for Environmental Medicine, Asthma and Lung Biology, University of North Carolina, 27599-7310, USA; 9 Department of Physiology and Biophysics, University of Calgary, Canada; 10 Institute for Inhalation Biology, GSF-National Research Center for Environment and Health, Neuherberg, Germany

## Abstract

Particulate air pollution has been associated with respiratory and cardiovascular disease. Evidence for cardiovascular and neurodegenerative effects of ambient particles was reviewed as part of a workshop. The purpose of this critical update is to summarize the evidence presented for the mechanisms involved in the translocation of particles from the lung to other organs and to highlight the potential of particles to cause neurodegenerative effects.

Fine and ultrafine particles, after deposition on the surfactant film at the air-liquid interface, are displaced by surface forces exerted on them by surfactant film and may then interact with primary target cells upon this displacement. Ultrafine and fine particles can then penetrate through the different tissue compartments of the lungs and eventually reach the capillaries and circulating cells or constituents, e.g. erythrocytes. These particles are then translocated by the circulation to other organs including the liver, the spleen, the kidneys, the heart and the brain, where they may be deposited. It remains to be shown by which mechanisms ultrafine particles penetrate through pulmonary tissue and enter capillaries. In addition to translocation of ultrafine particles through the tissue, fine and coarse particles may be phagocytized by macrophages and dendritic cells which may carry the particles to lymph nodes in the lung or to those closely associated with the lungs. There is the potential for neurodegenerative consequence of particle entry to the brain. Histological evidence of neurodegeneration has been reported in both canine and human brains exposed to high ambient PM levels, suggesting the potential for neurotoxic consequences of PM-CNS entry. PM mediated damage may be caused by the oxidative stress pathway. Thus, oxidative stress due to nutrition, age, genetics among others may increase the susceptibility for neurodegenerative diseases. The relationship between PM exposure and CNS degeneration can also be detected under controlled experimental conditions. Transgenic mice (Apo E -/-), known to have high base line levels of oxidative stress, were exposed by inhalation to well characterized, concentrated ambient air pollution. Morphometric analysis of the CNS indicated unequivocally that the brain is a critical target for PM exposure and implicated oxidative stress as a predisposing factor that links PM exposure and susceptibility to neurodegeneration.

Together, these data present evidence for potential translocation of ambient particles on organs distant from the lung and the neurodegenerative consequences of exposure to air pollutants.

## Background

Particulate air pollution has been associated with acute and chronic health effects due to respiratory and cardiovascular disease exacerbation [1,2]. New air quality standards have been introduced in Europe to control particulate matter with an aerodynamic diameter less then 10 μm (PM_10_) [3]. However, particles in ambient air are a complex mixture and might be quite diverse in terms of their chemical composition and their sizes. In particular, ultrafine particles (i.e., particles with diameters <100 nm) represent a class of particles with health effects independent of PM_10 _[4]. Their nanosize results in high particle density concentrations and large surface areas, relative to their mass.

The cardiovascular and neurological effects of fine and ultrafine particulate matter (PM) were discussed as part of a workshop held in Munich in August 2004. The symposium was hosted by the GSF-National Research Center for Environment and Health, the Research Focus Network Health Effects of Aerosols and the HGF Environmental Health Thematic Network. The symposium was organized by Annette Peters, Institute of Epidemiology and Holger Schulz, Institute for Inhalation Biology. The symposium highlighted recent advancements in addressing the health effects of fine and ultrafine particles on the cardiovascular system as reviewed recently [2,5,6].

Evidence describing particle translocation from the lungs has recently been summarized [7]. Interest in the non-pulmonary targets of particulate air pollutants has been increasing since the demonstration by Kreyling and others that inhaled, nanosize particles quickly left the lungs and were deposited in extra-pulmonary tissues [7,8]. The possibility that the brain was a potential target was raised in a 2002 editorial [9].

Experimental data was presented on how particles could translocate from their initial pulmonary target site into systemic circulation, and ultimately deposit in other organ systems (e.g., brain). Further in two presentations clinical and experimental evidence was given for neurodegeneration following PM exposure. The present critical update discusses the evidence for potential mechanisms for translocation of particles and their effects on the brain.

### Translocation of fine and ultrafine particles in the lung

With each breath millions of particles enter the lungs, where they may land on the surface of the airways or the alveoli. Upon deposition, the processes of retention and clearance begin. The fate of these particles depends on a number of factors particularly the particle size but also the anatomical location of the deposition in the airways or alveoli, and the histological structures the particles interact with at the site of deposition. The surfactant film is the first structure encountered by the deposited particles. This film lies at the air-aqueous liquid interface and covers the whole internal surface of the respiratory tract. After particles deposit on the surfactant film, they are wetted and displaced toward the epithelium by the surface forces generated at the interfaces between the air, particle and liquid [10,11]. In addition to the surface tension forces, line tension at the 3-phase line between the particle, air and liquid is likely important for particle wetting and displacement. Surface and line tension forces depend on the interfacial properties of the interacting systems, including the particles themselves, the air and the surrounding aqueous medium, with the interfacial film between medium and particle [12]. Particle displacement is the first step in the movement of the particles towards the walls of the airways and alveoli. It has been shown that smaller particles are more effectively displaced by a fluid phase covered with a surfactant film than larger particles [10,11]. However, all particles which penetrate the lung (PM_10_) are displaced by surface forces regardless of their characteristics [13]. After deposition, other particle characteristics such as shape, surface chemistry and the solubility of the particles largely determine their fate. Of particular importance for the generation of lung disease is the residence time of the particles in the respiratory tract.

**Figure 1:**
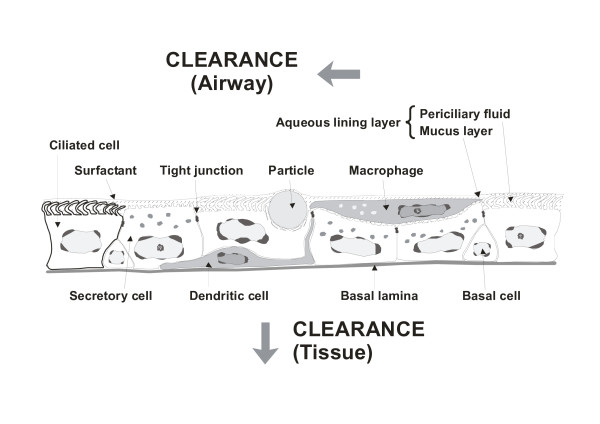
Schematic drawing of airway epithelial barrier with macrophages and dendritic cells, exposed to a fine particle (redrawn after McWilliam et al., 2000).

These surface and line tension forces determine whether or not the particles are brought into close proximity with the epithelial cells and the cells of the defence system, including the macrophages on the epithelial layer, or with dendritic cells at the base of the epithelium. Dendritic cells push fine cytoplasmic processes up to the tight junctions between the epithelial cells. (Figure 1, [14]). The particle-cell interactions that result from the forces associated with the free energy of interfaces and dividing lines may have implications for particle pathogenicity and the persistence of small particles, in particular, i.e. ultrafine particles or nanoparticles [15].

We have postulated that during the initial step of retention, the particle may adsorb components of the surfactant film or are coated by it. This particle modification may determine whether the particles are carried away free or in macrophages up the airways by the mucociliary action, or whether they are translocated into the tissue via dendritic cells. These cells may process the particles and carry them to the specific immunological defence system, that is the particles are presented to T-lymphocytes where they eventually initiate an immune reaction [16]. The dendritic cells are called "sentinels" of the pulmonary immune system because of their crucial role in pulmonary defence [14,17]. In contrast to macrophages, which engulf as much material as possible for efficient clearance, dendritic cells probably take up only as much antigen material as necessary to stimulate an immune reaction with the T-cells. The dendritic cells may cooperate with macrophages on the luminal side of the epithelium and with the epithelial cells themselves. This mechanism may be important for the trans-epithelial translocation of particles.

The surfactant film may primarily function as a defence barrier, as its initial interaction with the particles is the first step of a complex immunological cascade in the lung. Particles are probably rendered less toxic and more attractive to phagocytic cells because of their modification by the surfactant film or its components. Hence, we have proposed that this modification is important for guiding the particles along that clearance pathway which is most beneficial for our health [16].

We have learned from epidemiologic studies hat ultrafine particles may have a toxicological role [18]. They may induce inflammatory and pro-thrombotic responses in the organism, which could promote atherosclerosis, thrombogenesis and the occurrence of cardiovascular events. Furthermore, they may directly act on cells in various organs inducing oxidative stress and eventually heritable mutations. They may also affect the autonomic nervous system [5,19,20].

Whereas the uptake of fine particles (0.1–2.5 μm in diameter) by macrophages is a specific ligand-receptor mediated actin based process (phagocytosis) the uptake of ultrafine particles (<0.1 μm in diameter) apparently occurs by other, non-specific mechanisms. These mechanisms include electrostatic, van der Waals and steric interactions: They are subsumed under the term "adhesive interaction" [21].

In order to study the non-specific mechanisms of particle uptake macrophages in culture have been exposed to fluorescent polystyrene test particles of different sizes, 1 μm, 0.2 μm and 0.078 μm. In addition, the macrophages, after having been treated with catochalasin D which depolymerises the actin network, have been exposed to the same particle types. The actin-myosin sliding filament system is one of the intracellular motility systems. The cell cultures were investigated under a confocal laser scanning light microscope Zeiss 510 META. The largest particles were taken up by a large fraction of the cultured macrophages (56% of the cells contained particles, SD 30%), but after treatment with cytochalasin D this fraction was much smaller (5%, 3%). There were only few macrophages (21%, 11%) with particles of 0.2 μm in diameter, and there were not less macrophages with particles after cytochalasin D treatment (20%, 10%). However, there was a substantial number of cells with ultrafine particles, with (77%, 15%) and without (80%, 15%) catochalasin D. These observations indicate that there is a particular threshold in particle size with respect to particle uptake. Particles with a diameter of 0.2 μm and smaller appear to enter cells passively, that is by a mechanism which is different from phagocytosis. Larger particles are much more avidly taken up by macrophages, but by the specific receptor mediated, actin-dependent mechanism. Below the particle size of 0.2 μm an increasing number of particles enter the macrophages, but by the non specific mechanisms mentioned above.

In order to elucidate the mechanisms regarding the passive uptake of particles, we conducted a control experiment with erythrocytes. Erythrocytes do not possess receptors at their outer surface and no intracellular actin-myosin system. Therefore, erythrocytes are not expected to take up particles. The exposure of erythrocytes to the same particle types as in the previous experiments showed that the 1 μm particles were not taken up at all, as expected, but particles of smaller size entered the cells passively and rapidly. This was confirmed by electron microscopy with 0.2 μm polystyrene and 0.02 μm TiO_2 _particles.

In additional experiments we conducted an inhalation study with rats [22]. The lungs of rats which had inhaled an aerosol of 0.02 μm TiO_2 _particles showed the following results. (1) The particles could be found in all lung compartments 1 hour after inhalation, and the amount of the particles found in a particular compartment was proportional to the volume fraction of that compartment in the lung. (2) The particle distribution was unchanged after 24 hours. (3) Some particles were found in erythrocytes within the pulmonary capillaries.

These experiments demonstrated that ultrafine particles, after deposition, are rapidly distributed into all tissue compartments of the lung, and that these particles may move between the tissue compartments. The particles eventually reach the capillary lumen and may penetrate into circulating cells and constituents, e.g. erythrocytes. Thereby, they may be distributed into other organs of the body, such as the liver, the heart, the kidneys and even the brain [8,23]. However, in the present work we have demonstrated that the way particles can be translocated to other organs is by the circulating blood. It remains to be shown by which mechanisms ultrafine particles penetrate cellular membranes by non-specific means. We subsumed these non-specific mechanisms under the concept of "adhesive interactions", however, the exact mechanism remains to be determined [22]. Particles which penetrate into cells may reach specific organelles, they may reach the nucleus and they may induce an oxidative burst within the organelle's membranes where NADPH oxidases are located. In addition they may induce the release of inflammatory mediators and cytokines by the cells. All these ultrastructural and ultrafunctional changes may have potential health effects which deviate from "classical" inflammatory pathways and, therefore, should be investigated in great detail. These findings described above together with the observation of Oberdörster and colleagues, who found evidence of translocation of ultrafine particles into the brain [24] raises concern about the potential impact of particles on the brain.

Currently it is unknown why some studies did not show translocation from the lung to other organs [25,26]. However, there is the possibility that translocation of particles may display a high variability within the population and that factors such as diseases impact the translocation substantially. The health impact of long-term exposures to particles on the individual is likely to be determined by the number of particles and the amount of their surface components that are translocated from the lung into the body and by the velocity these particles and components are cleared.

### Are particles a risk factor for Alzheimer's disease?

The neuropathological effects of PM entry into the CNS tissues were first described in mongrel dogs [27]. In these studies, histological evidence of chronic brain inflammation and an acceleration of Alzheimer's disease-like pathology occurred, suggesting that the brain is also adversely affected by air pollutants. Residents of Mexico City, a metropolitan area with ozone and PM levels that exceed the US air quality standards, exhibit evidence of chronic inflammation of the upper and lower respiratory tracts, alterations in circulating inflammatory mediators [28] and breakdown of the respiratory epithelial barrier in the nose [29].

We conducted a study using autopsy brain samples from Mexican subjects, all lifelong residents of two large cities with severe air pollution, Mexico City and Monterrey, and five small cities with low levels of air pollution. Mexico City (MC) is a megacity with 20 million inhabitants, 3.5 million vehicles and extensive industrial activity [30]. Monterrey is the second largest industrial city in the country with 3.5 million residents and thousands of industries. Ozone (O_3_) and PM are the major air pollutants for both Mexico City and Monterrey. Bacterial lipopolysaccharides (LPS) and metals are major components of PM [30]. We studied 19 subjects (n: 9 from low polluted towns, n: 10 from Mexico City and Monterrey). Autopsies were performed 4.1 ± 1.3 h after death. All subjects were non-smokers. Inclusion criteria included: 1) access to recent complete clinical information within 3 months of death through medical records, interviews with private practitioners, and evaluation of job performance; 2) no evidence of neurological disease or cognitive abnormalities by medical history, and by complete neuropathological examination; 3) negative family history of dementia; 4) negative history of drug addictions, occupational exposures to potential neurotoxicants, recent vaccinations, and intake of vitamins, dietary or herbal supplements; and a 5) negative history of nonsteroidal anti-inflammatory drugs (NSAID) and steroidal compounds. The subjects had no clinical history or pathological evidence of short or long-term inflammatory processes, administration of anti-inflammatory drugs or hormones, or events such as cerebral ischemia, head trauma or epilepsy.

**Figure 2:**
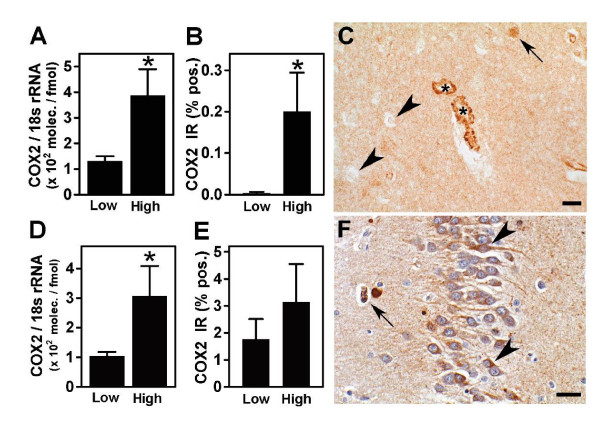
COX2 expression in frontal cortex and hippocampus. (A and D) COX2 mRNA abundance was measured by RT-PCR and normalized for 18s rRNA levels. Means ± SEMs are shown. COX2 mRNA was significantly elevated in the high exposure group in both frontal cortex (A, * p = 0.009) and hippocampus (D, * p = 0.04) from the high exposure group. (B and E) COX2 protein expression in sections of paraffin-embedded tissues was localized by COX2 immunohistochemistry (IHC) and the percent of tissue area that was immunoreactive (COX2 IR) was measured by quantitative image analysis. Means ± SEMs are shown. COX2 IR was significantly elevated in frontal cortex (B, * p = 0.01), but not in hippocampus (E) from the high exposure group. (C) Representative COX IHC in frontal cortex from a subject in the high exposure group showing strong staining of endothelial cells in the capillaries (*), and pyramidal neurons (arrow), while other neurons were negative (arrowheads). Scale = 20 μm. (F) Representative COX IHC in dentate gyrus from a subject in the high exposure group showing COX2 positive neurons (arrowheads) and capillaries (short arrow). Scale = 15 μm.

We selected COX2 mRNA levels as the primary variable because of the striking upregulation of COX2 immunoreactivity (IR) observed in the brains of dogs residing in Mexico City [27,31] and because neuronal COX2 expression in the hippocampus is a function of the clinical progression of Alzheimer's disease (AD) [32]. COX2 mRNA abundance was measured by real-time RT-PCR analysis of total RNA isolated from frontal cortex and hippocampus. In the 19 subjects for which frontal cortex tissue was available there was an elevation of COX2 mRNA levels in the high exposure group (p = 0.009, Mann-Whitney test) (Figure 2A). There was an elevation of COX2 IR in the high exposure group confirmed by quantitative image analysis of COX2 IR (p = 0.01, Mann-Whitney test) (Figure 2B). In the low exposure group, COX2 IR was confined to neuronal cell bodies, whereas subjects from the high exposure group exhibited strong COX2 staining of the endothelium both in the cortex (Figure 2C) and white matter blood vessels. In the 15 subjects for which hippocampus tissue was available, COX2 mRNA was also elevated in the high exposure group (p = 0.045, Mann-Whitney test) (Figure 2D). However, the average density of COX2 IR was not significantly different from levels in low exposure group (p = 0.3) (Figure 2E). Hippocampus COX2 IR in highly exposed subjects was observed in neuronal bodies included in the CA1–2 and CA4 regions and the dentate gyrus, as well as endothelial cells (Figure 2F).

**Figure 3:**
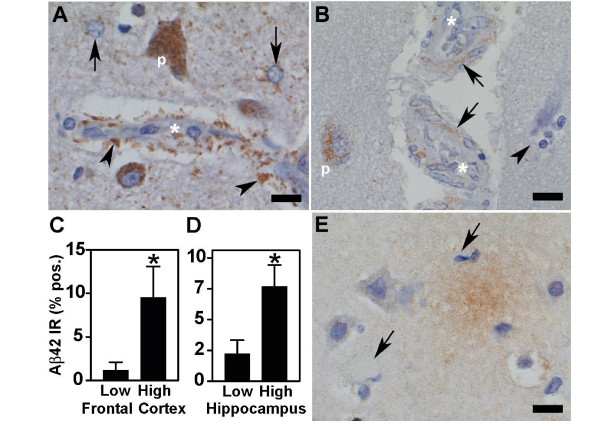
Aβ42 accumulation in frontal cortex and hippocampus. Aβ42 was localized in sections of paraffin-embedded tissues by IHC. (A) Aβ42 IHC stained pyramidal neurons (p), astrocytes (arrows) and astrocytic processes (arrowheads) around blood vessels (*). (B) In addition to accumulation in pyramidal neurons (p) Aβ42 was deposited in smooth muscle cells (arrows) in cortical arterioles (*). A dead neuron surrounded by glial cells is indicated (arrowhead). (C and D) Quantitative image analysis of Aβ42 IHC showed a significant increase in Aβ42 immunoreactivity (Aβ42 IR) in both frontal cortex (C, * p = 0.04) and hippocampus (D, * p = 0.001) in the high exposure group. (E) Aβ42 IHC of frontal cortex from a 38 year old subject from Mexico City showing diffuse plaque-like staining with surrounding reactive astrocytes (arrows). Scale = 20 μm.

Aβ42 accumulation was examined in frontal cortex and hippocampus because upregulation of COX2 expression in dogs is associated with Aβ42 accumulation [31], and because of reports of significant correlations between elevated levels of Aβ42 and cognitive decline [33]. In subjects from the high exposure group, Aβ42 selectively accumulated in the perikaryon of pyramidal frontal neurons as discrete granules and was present in cortical and white matter astrocytes, and subarachnoid and cortical blood vessels (Figure 3A and 3B). Quantitative estimation of Aβ42 IR confirmed an increase in Aβ42 accumulation in the frontal cortex (p = 0.04, Mann-Whitney test) (Figure 3C), and in hippocampus (p = 0.001, Mann-Whitney test) (Figure 3D) of the high exposure group. Rare diffuse Aβ42 plaque-like staining was present in the frontal cortex of 3 subjects in the high exposure group (32, 38 and 43 years old) (Figure 3E). The diffuse plaque-like staining was not associated with the apolipoprotein E ε4 allele.

COX2 and Aβ42 IR were present in the olfactory bulb from 3 subjects in the high exposure group and absent in 2 subjects from the low exposure group. The olfactory bulb is the first synaptic relay of neurons that reside in the olfactory epithelium and that are directly exposed to air pollutants. Olfactory neurons are capable of taking up and transporting environmental agents into the brain [31,34]. The presence of olfactory bulb pathology in subjects from the high exposure group suggests that the human nose may be a portal of entry of air pollutants into the brain [31].

Both chronic respiratory tract inflammation and breakdown of the nasal respiratory and olfactory barriers may contribute to brain inflammation [31]. Respiratory tract inflammation may play a role by chronically increasing the levels of circulating cytokines that can cross the blood brain barrier and evoke an inflammatory response [35]. Increased concentrations of pro- and anti-inflammatory serum cytokines are seen in Mexico City children along with evidence of significant respiratory tract damage, and breakdown of their nasal epithelial barriers [28,29]. Breakdown of epithelial barriers in the nose may contribute to brain inflammation by increasing the access of air pollutants to the brain [31]. Although the subjects in the high exposure group are chronically exposed to a complex mixture of air pollutants, a variety of evidence suggests that PM may play a role in the neuropathological findings reported here. PM contains bacterial lipopolysaccharide and combustion-derived metals such as nickel and vanadium [36] – all agents that are known to evoke inflammatory responses [31,36]. Ultrafine (< 100 nm) PM, when deposited in the lung, causes notable inflammation [37], and ultrafine PM can be transported into the systemic circulation [38]. A recent study shows that tobacco smoking, a behavior that involves chronic high dose PM exposures, is associated with a dose-dependent increase in the risk of developing AD later in life [39], supporting an association between PM exposure and neurological effects. The pathology observed in the subjects chronically exposed to severe air pollution has a number of similarities to the pathology of AD. COX2 expression is elevated in the early stages of Alzheimer's disease [40], intraneuronal Aβ42 accumulation in target areas precedes Aβ plaque deposition and neurofibrillary tangles [41-43], and olfactory bulb pathology similar to that observed in the high exposure group is one of the earliest pathological findings in Alzheimer's disease [44], where it is associated with impaired olfaction. The identification of COX2 upregulation as an early marker of neuroinflammation in the context of air pollutant exposure suggests that nonsteroidal anti-inflammatory drugs (NSAIDs) may ameliorate the neuropathological effects of exposure to air pollutants. Our findings suggest that exposure to severe urban air pollution is associated with brain inflammation and amyloid deposits, causes of neuronal dysfunction that precede the appearance of neuritic plaque formation and neurofibrillary tangles, hallmarks of Alzheimer's disease.

These findings in highly exposed individuals raise a crucial issue: the role environment and air pollutants in particular play in the pathogenesis of neurodegenerative diseases such as Alzheimer's. Although the number of subjects was small due to the strict inclusion criteria, the findings of this study concur with the acceleration of Alzheimer's type pathology in Mexico City dogs [27,31]. These studies warrant additional forensic and epidemiological studies supporting the association between chronic exposure to air pollutants and the development of Alzheimer's disease.

Currently, Alzheimer's disease is an irreversible, fatal brain disorder that diminishes the quality of life of affected individuals and places a burden on the health care system [45]. In 2000 there were 4.5 million people with AD in the US and it is projected that AD will affect 13.2 million by 2050 [45]. The role played by the environment in the pathogenesis of AD is unknown.

### Neurodegeneration in Apo E-/- mice exposed to concentrated ambient particulate matter: influence of oxidative stress

A study was designed to assess the long-term impact of concentrated ambient particles on various organ systems of mice [46,47]. Detailed descriptions were published of the experimental design [47] and modification of the exposure system [48]. The observed effects ranged from changes in heart rate (HR), heart rate variability (HRV), atherosclerotic plaques on endothelia, gene expression, and brain cell distributions [49-53].

In this study, histological changes suggestive of oxidative stress-mediated damage were described including disruption of the blood-brain barrier, degeneration of cortical neurons, apoptotic glial cells, and neurofibrillary tangles and increases in NFκβ. Shortly after these reports were published, we initiated an experimental study to determine if animals, transgenically predisposed to oxidative stress (OS), would express neurodegeneration in response to concentrated ambient particles (CAPs). A more extensive description of this pathology and companion studies on the enhanced cardiopulmonary toxicity have since been published [53]. The Apo E deficient "knock out" mouse (Apo E^-/-^) was used in this study because of numerous reports describing heightened levels of OS and errors in modulating OS in the brain [54-57]. Apo E^-/- ^mice and those from the normal C57BL/6 background strain, were obtained commercially (Jackson Laboratory, Bar Harbor, ME, USA) and exposed for 5 mo to concentrated northeastern regional (Tuxedo, NY, USA) ambient particles (CAPs).

A cohort of C57BL/6 (C57) mice was used to investigate effects on the respiratory system. Other cohorts, i.e. C57 (n = 6), and ApoE^-/- ^mice, implanted with ECG transmitters (DataScience), were used to investigate the effects of CAPs on the cardiovascular system. A separate cohort of double knockout mice (DK, ApoE and LDLr knockout) was also included to investigate histopathological changes and gene expression patterns of the cardiovascular and pulmonary systems. The overall mean concentration during the 30 hr/week CAPs exposure was 110 ± 79 μg/m^3 ^(19.6 μg/m^3 ^normalized annually). A modified VACES [48] was used to expose mice at NYU's Sterling Forest Laboratory to a ten-fold concentration of Northeastern regional background CAPs daily for 6 hr/d, 5 d/wk between Mar. and Sept. 2003.

#### Evidence for changes in autonomic function

A recently developed non-parametric statistical method [58] was used to estimate the times that mean heart rates, body temperature, and physical activity differed significantly between the CAPs and sham exposed groups. CAPs exposure most affected HR between 1:30 a.m. – 4:30 a.m., and the greatest effects were seen at the end of the 5-month exposure. A two-stage modeling approach was used to obtain the estimates of chronic and acute effects on these three response variables. There were significant decreasing patterns of HR (reaching a reduction of 33.8 beats/min in HR in CAPs exposed ApoE^-/-^mice compared to air exposed ApoE^-/- ^controls), body temperature, and physical activity in ApoE^-/- ^mice over the five months of CAPs exposure, with smaller and non-significant changes in C57 mice [52]. In addition, there was a 10 beats/min per 100 μg/m^3 ^decrease in HR during the exposure period for the ApoE^-/- ^mice that was not seen in the C57 mice.

At the same time, there was a quite different pattern of change for HRV (SDNN and RMSSD) [50]. There was a prolonged elevation, peaking at about two months into the study, a decline to below the initial levels by 4 months, and a relatively modest change in the last month of the exposure series. There were no effects seen in normal C57 mice exposed to the same atmospheres. The response patterns indicated a perturbation of the homeostatic function in the cardiovascular system with initial stimulation (enhancement) and later depression of the HRV parameters.

#### Evidence for progression of atherosclerosis

The lungs, the hearts, the aortas, the brains, and the upper airways of all mice were harvested for histopathological examination. The cross sectional area of the aorta root of DK mice was examined morphologically using confocal microscopy for the severity of lesion, extent of cellularity, and lipid contents. Aortas from the arch to the iliac bifurcations were also sectioned longitudinally and lesion areas were stained with Sudan IV [49]. All DK mice, regardless of exposure, had developed extensive lesions in the aortic sinus regions, with lesion areas that covered more than 79% of the total area. In male DK mice, the lesion areas in the aortic sinus regions appeared to be enhanced by CAPs, with changes approaching statistical significance (p = 0.06). In addition, plaque cellularity was increased by 28% (p = 0.014, combined) whereas there was no CAPs associated changes in the lipid content in these mice.

When examining the entire aorta opened longitudinally, both the ApoE^-/- ^and DK mice had prominent areas of severe atherosclerosis covering 40% or more of the lumenal surface. Visual examination of all images suggested that plaques tend to form in clusters concentrating near the aortic arch and the iliac bifurcations. Quantitative measurements showed that CAPs exposure increased the percentage of aortic intimal surface covered by grossly discernible atherosclerotic lesion by 57% in the ApoE^-/- ^mice (p = 0.03). Changes produced by CAPS in male (10% increase) or female DK mice (8% decrease) were not statistically significant. Thus, subchronic exposure to CAPs in mice prone to develop atherosclerotic lesions had a significant impact on the size, severity, and composition of aortic plaque. Effects of CAPs on non-susceptible C57 were minimal.

#### Evidence for oxidative stress in the brains

**Figure 4:**
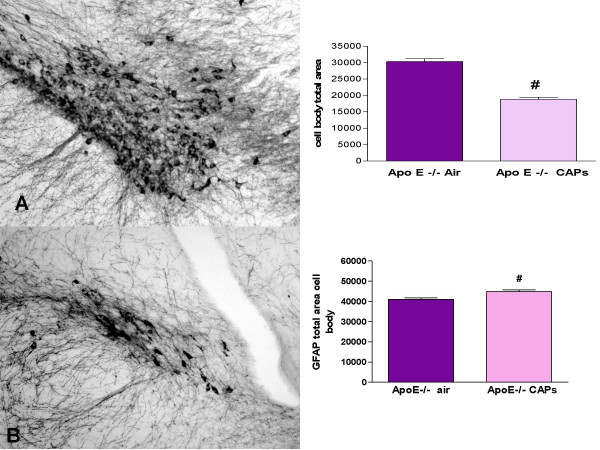
Micrographs of the SN of (A) Air and (B) CAPs exposed Apo E -/- mouse brain and representative examples of the nucleus compacta which house the dopaminergic neurons, vulnerable to OS. Sections were taken from the same brain level. Neurons and astrocytes were immunocytochemical stained with TH and GFAP, respectively. A 29% reduction of TH staining and an 8% increase in GFAP was measured in the Apo E-/- PM exposed mice relative to air exposed Apo E-/- No differences in TH or GFAP bodies were observed in C57 Air or PM exposed animals. Sections 300× magnification.

**Figure 5:**
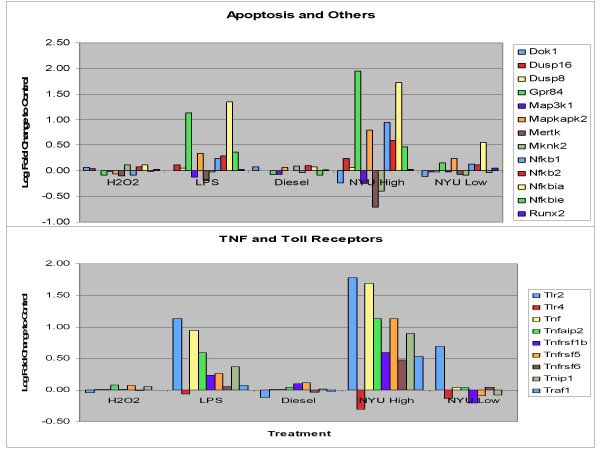
Genomics: BV2 microglia were exposed in triplicate (2 hr) to CAPs (75 μg/ml), and other chemicals known to produce OS (i.e., LPS (2.5 ng/ml); H202 (0.2 mM), Diesel exhaust filtrate (100 μg/ml). RNAs from each test group were analyzed on Affymetrix universal microarrays. Relative to the other treatment and controls, CAPs produced significant increases/decreases in genes coding for apoptotic, TNF and Toll receptors; cytokines, interferons, kinase transcription, oncogenes and growth factors.

The brains of CAPs and air exposed Apo E^-/- ^mice (6–9/treatment) and C57BL/6 (5/treatment) were multi-embedded (25 per slide), and serially sectioned in the coronal plane. Slides were immunohistologically stained for tyrosine hydroxylase (TH), a marker for dopamine containing neurons and glial fibrillary acidic protein (GFAP), a marker of astrocytic proliferation. The nuclei reticularis and compacta of the substantia nigra (SN) are anatomical regions which house dopaminergic neurons, a population particularly sensitive to OS and predominantly damaged in human and animal models of Parkinson's neuropathy [59]. These regions were photographed with a cool frame, Hamamatsu camera, digitized and morphometrically analyzed by counting the total pixel stained area of TH or GFAP stained cell bodies. Results showed that quantitative analysis of the TH-stained neurons and their fiber plexus showed no significant differences in the air or CAPs of exposed C57/blk6 relative to the air exposed Apo E -/- animals. However, there was a 29% reduction in TH stained neurons in the CAPs exposed relative to air exposed Apo E -/- mice (Figure 4). In addition, a significant 8% increase (p < 0.05) in GFAP staining (i.e., astrocytes) was measured in the nucleus compacta of CAPs exposed Apo E -/- relative to air exposed Apo E -/- brains. To examine possible mechanisms behind the neurodegeneration seen in the CAPs animals described above, immortalized mouse microglia (BV2) cells were exposed to the same CAPs and examined for cellular and genomic expressions of OS. The cultured microglia responded with an initial oxidative burst and mitochondrial depolarization, followed by a release of inflammatory cytokines [53]. Universal microarrays of microglial RNA indicated numerous OS pathways (Nfkb, Toll receptor, glutathione etc.) were up-regulated after 2 hr exposure (Figure 5).

The present data suggest that controlled exposure to concentrated ambient air produces significant neurodegeneration in an animal model predisposed to OS (i.e., Apo E -/-). The presence of OS markers in the CNS tissues of victims with amyolateral sclerosis, Parkinson's and Alzheimer's has also been reported [60,61]. The possibility that such neurodegenerative disorders may have an environmental component cannot be excluded, especially in view of the OS associated with certain environmental pollutants (e.g., pesticides, metals, dioxides, PM). It is known that PM particles are covered with biocontaminants and other organic pollutants that contribute to the free radical activity carried on the particles' surface. Contact with such particles can damage lipids, nucleic acids, and proteins at deposition sites within the lungs and in secondary targets. The brain is vulnerable to OS because of its high energy demands, low levels of endogenous scavengers (e.g., vitamin C, catalase, superoxide dismutase, etc.), and high cellular content of lipids and proteins. Certain neuronal populations (such as the SN dopaminergic neurons) are especially vulnerable to OS damage since they have heightened energy demands due to their extensive synaptic fields and length of their fiber tracts [62]. The affected populations in the present study were the dopaminergic populations of the nigrostriatal pathway, the same population targeted in Parkinson's disease [62,63].

Incidences of neurodegenerative diseases appear to be increasing within the population [64] and raise the possibility that environmental exposures, combined with susceptibility factors (e.g., genetics, diseased states, malnutrition, etc.), might contribute to these increases. Due to our cell culture data, we are confident to propose that PM induced neurodegeneration is mediated by CNS macrophages (i.e., microglia). The released reactive oxygen species, which remain active in OS "compromised" individuals, will in turn, damage OS vulnerable neurons located in their microenvironment. In support of this is a recent study which describes microglia-mediated, selective neurodegeneration to the dopaminergic neurons in a primary CNS cultures exposed to nanometer sized particles from diesel engine exhaust [65].

Although it is possible that PM nanosize particles enter the CNS olfactory bulb via olfactory tubercles, the biological barriers (e.g., CO_2_:O_2 _interface, blood brain barrier routes) encountered by the particles exiting the lungs and entering the CNS are the more likely routes of entry to the CNS neuropile. Damage to these endothelial barriers by the free radical activity carried on the PM particle's surface would disrupt the tight junctions and facilitate particle translocation. In addition, new data suggest that PM neurodegeneration might be initiated by innate immunity pathways. Through such processes, inflammatory cytokines, released in the periphery (e.g., respiratory epithelia), can increase CNS levels of NF-κB activation and up-regulate the innate immune receptor Toll-like receptor 2. Such activation and the subsequent events leading to neurodegeneration have recently been described in BALB/c mice exposed to ambient Los Angeles particulate matter [66]. Such data indicate a neuroimmunological pathway to explain PM-CNS neurodegeneration. Together, these reports confirm that the brain is a critical target of PM exposure and implicate OS as a predisposing factor that links PM exposure and neurotoxic susceptibility.

## Conclusion

The data discussed as part of this critical update highlights that ultrafine particles rapidly translocate from the lungs into the cells and in particular into the blood. There is mounting evidence that signs of oxidative stress can be found in other organs, such as the heart [67] or the brain. The results indicating that particles may contribute to the overall oxidative stress burden of the brain is particularly troublesome, as these long-term health effects may accumulate over decades. The characteristics and sources of particles responsible for the oxidative stress burden need to be identified. In addition, further studies are needed in humans to verify and quantify the relative risks for long-term particle exposure on the onset of Parkinson's and Alzheimer's disease. Both Parkinson's and Alzheimer's disease are only diagnosed once manifest clinical signs and symptoms are evident and impact the diseased persons by long years of disabilities and diminished quality of life.

## Competing interests

The author(s) declare that they have no competing interests.

## Authors' contributions

AP and HS have made substantial contributions to conception and design of the article, BV, LCC, LCC, PG, MGK, WR, BRR, SS conceived the studies described, acquired, analysed and interpreted the data, 2) all authors have been involved in drafting the manuscript or revising it critically for important intellectual content; and 3) all authors have given final approval of the version to be published.

## References

[B1] Brunekreef B, Holgate ST (2002). Air pollution and health. Lancet.

[B2] Brook RD, Franklin B, Cascio WE, Hong Y, Howard G, Lipsett M, Luepker RV, Mittleman MA, Samet JM, Smith SCJ, Tager IB (2004). Air Pollution and Cardiovascular Disease: A statement of the health care professionals from the expert panel on population and prevention science of the American Heart Association. Circulation.

[B3] Kappos AD, Bruckmann P, Eikmann T, Englert N, Heinrich U, Hoppe P, Koch E, Krause GH, Kreyling WG, Rauchfuss K, Rombout P, Schulz-Klemp V, Thiel WR, Wichmann HE (2004). Health effects of particles in ambient air. Int J Hyg Environ Health.

[B4] Ibald-Mulli A, Wichmann H, Kreyling WG, Peters A (2002). Epidemiological Evidence on Health Effects of Ultrafine Particles. Journal of Aerosol Medicine.

[B5] Schulz H, Harder V, Ibald-Mulli A, Khandoga A, Koenig W, Krombach F, Radykewicz R, Stampfl A, Thorand B, Peters A (2005). Cardiovascular effects of fine and ultrafine particles. J Aerosol Med.

[B6] Peters A (2005). Particulate matter and heart disease: Evidence from epidemiological studies. Toxicol Appl Pharmacol.

[B7] Kreyling WG, Semmler-Behnke M, Moller W (2006). Ultrafine particle-lung interactions: does size matter?. J Aerosol Med.

[B8] Kreyling WG, Semmler M, Erbe F, Mayer P, Takenaka S, Schulz H, Oberdorster G, Ziesenis A (2002). Translocation of ultrafine insoluble iridium particles from lung epithelium to extrapulmonary organs is size dependent but very low. J Toxicol Environ Health A.

[B9] Oberdorster G, Utell MJ (2002). Ultrafine particles in the urban air: to the respiratory tract--and beyond?. Environ.

[B10] Gehr P, Schürch S, Berthiaume Y, Im Hof V, Geiser M (1996). Particle retention in airways by surfactant. J Aerosol Med.

[B11] Schurch S, Gehr P, Im HV, Geiser M, Green F (1990). Surfactant displaces particles toward the epithelium in airways and alveoli. Respir Physiol.

[B12] Gehr P, Geiser M, Im Hof V, Schürch S (2000). Surfactant-ultrafine particle interactions: what we can learn from PM10 studies. Phil Trans R Soc Lond A.

[B13] Geiser M, Schurch S, Gehr P (2003). Influence of surface chemistry and topography of particles on their immersion into the lung's surface-lining layer. J Appl Physiol.

[B14] McWilliam AS, Holt PG, Gehr P (2000). Dendritic cells as sentinels of immune surveillance in the airways. In: Particle-Lung Interaction. Lung Biology in Health and Disease.

[B15] Oberdorster G, Ferin J, Lehnert BE (1994). Correlation between particle size, in vivo particle persistence, and lung injury. Environ.

[B16] Gehr P, Green FH, Geiser M, Im HV, Lee MM, Schurch S (1996). Airway surfactant, a primary defense barrier: mechanical and immunological aspects. J Aerosol Med.

[B17] Holt PG, Schon-Hegrad MA (1987). Localization of T cells, macrophages and dendritic cells in rat respiratory tract tissue: implications for immune function studies. Immunology.

[B18] Peters A, Wichmann HE, Tuch T, Heinrich J, Heyder J (1997). Respiratory effects are associated with the number of ultra-fine particles. Am J Respir Crit Care Med.

[B19] Choi JH, Kim JS, Kim YC, Kim YS, Chung NH, Cho MH (2004). Comparative study of PM2.5 - and PM10 induced OS in rat lung epithelial cells. J Vet Sci.

[B20] Samet JM, DeMarini DM, Malling HV (2004). Biomedicine. Do airborne particles induce heritable mutations?. Science.

[B21] Rimai DSQDJBAA (2000). The adhesion of dry particles in the nanometer to micrometer-size range. Coll Surf A (Physiocochemical and Engineering Aspects).

[B22] Geiser M, Rothen-Rutishauser B, Kapp N, Schurch S, Kreyling W, Schulz H, Semmler M, Im HV, Heyder J, Gehr P (2005). Ultrafine particles cross cellular membranes by nonphagocytic mechanisms in lungs and in cultured cells. Environ Health Perspect.

[B23] Oberdorster G, Sharp Z, Atudorei V, Elder A, Gelein R, Lunts A, Kreyling W, Cox C (2002). Extrapulmonary translocation of ultrafine carbon particles following whole-body inhalation exposure of rats. J Toxicol Environ Health A.

[B24] Oberdorster G, Sharp Z, Atudorei V, Elder A, Gelein R, Kreyling W, Cox C (2004). Translocation of inhaled ultrafine particles to the brain. Inhal Toxicol.

[B25] Brown JS, Zeman KL, Bennett WD (2002). Ultrafine particle deposition and clearance in the healthy and obstructed lung. Am J Respir Crit Care Med.

[B26] Mills NL, Amin N, Robinson SD, Anand A, Davies J, Patel D, de la Fuente JM, Cassee FR, Boon NA, MacNee W, Millar AM, Donaldson K, Newby DE (2006). Do inhaled carbon nanoparticles translocate directly into the circulation in humans?. Am J Respir Crit Care Med.

[B27] Calderon-Garciduenas L, Azzarelli B, Acuna H, Garcia R, Gambling TM, Osnaya N, Monroy S, DEL Tizapantzi MR, Carson JL, Villarreal-Calderon A, Rewcastle B (2002). Air pollution and brain damage. Toxicol Pathol.

[B28] Calderon-Garciduenas L, Mora-Tiscareno A, Fordham LA, Valencia-Salazar G, Chung CJ, Rodriguez-Alcaraz A, Paredes R, Variakojis D, Villarreal-Calderon A, Flores-Camacho L, Antunez-Solis A, Henriquez-Roldan C, Hazucha MJ (2003). Respiratory damage in children exposed to urban pollution. Pediatr Pulmonol.

[B29] Calderon-Garciduenas L, Valencia-Salazar G, Rodriguez-Alcaraz A, Gambling TM, Garcia R, Osnaya N, Villarreal-Calderon A, Devlin RB, Carson JL (2001). Ultrastructural nasal pathology in children chronically and sequentially exposed to air pollutants. Am J Respir Cell Mol Biol.

[B30] Bravo HA, Torres RJ, Fenn M, Bauer L and Hernández T (2002). Air Pollution levels and trends in the Mexico City metropolitan area. In Urban air pollution and forests: resources at risk in the Mexico City Air Basin.

[B31] Calderon-Garciduenas L, Maronpot RR, Torres-Jardon R, Henriquez-Roldan C, Schoonhoven R, Acuna-Ayala H, Villarreal-Calderon A, Nakamura J, Fernando R, Reed W, Azzarelli B, Swenberg JA (2003). DNA damage in nasal and brain tissues of canines exposed to air pollutants is associated with evidence of chronic brain inflammation and neurodegeneration. Toxicol Pathol.

[B32] Ho L, Purohit D, Haroutunian V, Luterman JD, Willis F, Naslund J, Buxbaum JD, Mohs RC, Aisen PS, Pasinetti GM (2001). Neuronal cyclooxygenase 2 expression in the hippocampal formation as a function of the clinical progression of Alzheimer disease. Arch Neurol.

[B33] Naslund J, Haroutunian V, Mohs R, Davis KL, Davies P, Greengard P, Buxbaum JD (2000). Correlation between elevated levels of amyloid beta-peptide in the brain and cognitive decline. JAMA.

[B34] Dorman DC, Brenneman KA, McElveen AM, Lynch SE, Roberts KC, Wong BA (2002). Olfactory transport: a direct route of delivery of inhaled manganese phosphate to the rat brain. J Toxicol Environ Health A.

[B35] Rivest S (2001). How circulating cytokines trigger the neural circuits that control the hypothalamic-pituitary-adrenal axis. Psychoneuroendocrinology.

[B36] Elmquist JK, Breder CD, Sherin JE, Scammell TE, Hickey WF, Dewitt D, Saper CB (1997). Intravenous lipopolysaccharide induces cyclooxygenase 2-like immunoreactivity in rat brain perivascular microglia and meningeal macrophages. J Comp Neurol.

[B37] Oberdorster G (2001). Pulmonary effects of inhaled ultrafine particles. Int Arch Occup Environ Health.

[B38] Calderon-Garciduenas L, Mora-Tiscareno A, Fordham LA, Chung CJ, Garcia R, Osnaya N, Hernandez J, Acuna H, Gambling TM, Villarreal-Calderon A, Carson J, Koren HS, Devlin RB (2001). Canines as sentinel species for assessing chronic exposures to air pollutants: part 1. Respiratory pathology. Toxicol Sci.

[B39] Tyas SL, White LR, Petrovitch H, Webster RG, Foley DJ, Heimovitz HK, Launer LJ (2003). Mid-life smoking and late-life dementia: the Honolulu-Asia Aging Study. Neurobiol Aging.

[B40] Yermakova AV, O'Banion MK (2001). Downregulation of neuronal cyclooxygenase-2 expression in end stage Alzheimer's disease. Neurobiol Aging.

[B41] Gouras GK, Tsai J, Naslund J, Vincent B, Edgar M, Checler F, Greenfield JP, Haroutunian V, Buxbaum JD, Xu H, Greengard P, Relkin NR (2000). Intraneuronal Abeta42 accumulation in human brain. Am J Pathol.

[B42] Selkoe DJ (2001). Alzheimer's disease: genes, proteins, and therapy. Physiol Rev.

[B43] Gyure KA, Durham R, Stewart WF, Smialek JE, Troncoso JC (2001). Intraneuronal abeta-amyloid precedes development of amyloid plaques in Down syndrome. Arch Pathol Lab Med.

[B44] Braak H, de Vos RA, Jansen EN, Bratzke H, Braak E (1998). Neuropathological hallmarks of Alzheimer's and Parkinson's diseases. Prog Brain Res.

[B45] Hebert LE, Scherr PA, Bienias JL, Bennett DA, Evans DA (2003). Alzheimer disease in the US population: prevalence estimates using the 2000 census. Arch Neurol.

[B46] Lippmann M, Gordon T, Chen LC (2005). Effects of subchronic exposures to concentrated ambient particles in mice. IX. Integral assessment and human health implications of subchronic exposures of mice to CAPs. Inhal Toxicol.

[B47] Lippmann M, Gordon T, Chen LC (2005). Effects of subchronic exposures to concentrated ambient particles (CAPs) in mice. I. Introduction, objectives, and experimental plan. Inhal Toxicol.

[B48] Maciejczyk P, Zhong M, Li Q, Xiong J, Nadziejko C, Chen LC (2005). Effects of subchronic exposures to concentrated ambient particles (CAPs) in mice. II. The design of a CAPs exposure system for biometric telemetry monitoring. Inhal Toxicol.

[B49] Chen LC, Nadziejko C (2005). Effects of subchronic exposures to concentrated ambient particles (CAPs) in mice. V. CAPs exacerbate aortic plaque development in hyperlipidemic mice. Inhal Toxicol.

[B50] Chen LC, Hwang JS (2005). Effects of subchronic exposures to concentrated ambient particles (CAPs) in mice. IV. Characterization of acute and chronic effects of ambient air fine particulate matter exposures on heart-rate variability. Inhal Toxicol.

[B51] Gunnison A, Chen LC (2005). Effects of subchronic exposures to concentrated ambient particles (CAPs) in mice. VI. Gene expression in heart and lung tissue. Inhal Toxicol.

[B52] Hwang JS, Nadziejko C, Chen LC (2005). Effects of subchronic exposures to concentrated ambient particles (CAPs) in mice. III. Acute and chronic effects of CAPs on heart rate, heart-rate fluctuation, and body temperature. Inhal Toxicol.

[B53] Veronesi B, Makwana O, Pooler M, Chen LC (2005). Effects of subchronic exposures to concentrated ambient particles. VII. Degeneration of dopaminergic neurons in Apo E-/- mice. Inhal Toxicol.

[B54] Colton CA, Brown CM, Cook D, Needham LK, Xu Q, Czapiga M, Saunders AM, Schmechel DE, Rasheed K, Vitek MP (2002). APOE and the regulation of microglial nitric oxide production: a link between genetic risk and oxidative stress. Neurobiol Aging.

[B55] Law A, Gauthier S, Quirion R (2003). Alteration of nitric oxide synthase activity in young and aged apolipoprotein E-deficient mice. Neurobiol Aging.

[B56] Ramassamy C, Krzywkowski P, Averill D, Lussier-Cacan S, Theroux L, Christen Y, Davignon J, Poirier J (2001). Impact of apoE deficiency on oxidative insults and antioxidant levels in the brain. Brain Res Mol Brain Res.

[B57] Shea TB, Rogers E, Ashline D, Ortiz D, Sheu MS (2002). Apolipoprotein E deficiency promotes increased oxidative stress and compensatory increases in antioxidants in brain tissue. Free Radic Biol Med.

[B58] Nadziejko C, Chi CL, Nadas A, Hwang JS (2004). The 'Fishing License' method for analysing the time course of effects in repeated measurements. Stat Med.

[B59] Greenamyre JT, Betarbet R, Sherer TB (2003). The rotenone model of Parkinson's disease: genes, environment and mitochondria. Parkinsonism Relat Disord.

[B60] Halliwell B (2001). Role of free radicals in the neurodegenerative diseases: therapeutic implications for antioxidant treatment. Drugs Aging.

[B61] Mattson MP (2001). Mechanisms of neuronal apoptosis and excitotoxicity. Pathogenesis of neurodegenerative disorders..

[B62] Hirsch EC, Faucheux B, Damier P, Mouatt-Prigent A, Agid Y (1997). Neuronal vulnerability in Parkinson's disease. J Neural Transm Suppl.

[B63] Sherer TB, Betarbet R, Greenamyre JT (2002). Environment, mitochondria, and Parkinson's disease. Neuroscientist.

[B64] Fuente-Fernandez R, Calne DB (2002). Evidence for environmental causation of Parkinson's disease. Parkinsonism Relat Disord.

[B65] Block ML, Wu X, Pei Z, Li G, Wang T, Qin L, Wilson B, Yang J, Hong JS, Veronesi B (2004). Nanometer size diesel exhaust particles are selectively toxic to dopaminergic neurons: the role of microglia, phagocytosis, and NADPH oxidase. FASEB J.

[B66] Campbell A, Mendez L, Becaria AM, Kleinmann J (2006). Enhancement of innate immune responses and oxidative events after exposure to particulate matter. The Toxicologist.

[B67] Gurgueira SA, Lawrence J, Coull B, Murthy GG, Gonzalez-Flecha B (2002). Rapid increases in the steady-state concentration of reactive oxygen species in the lungs and heart after particulate air pollution inhalation. Environ Health Perspect.

